# Promoting the Brand Inside: The Conceptualization of Nonprofit Internal Branding and Its Relationship With Employees’ Brand Performance

**DOI:** 10.3389/fpsyg.2022.722057

**Published:** 2022-07-13

**Authors:** Ran Zhang, Yunqiao Wu, Chao Ye

**Affiliations:** ^1^School of Public Administration, East China Normal University, Shanghai, China; ^2^School of Business and Management, Shanghai International Studies University, Shanghai, China; ^3^School of Law, Shanghai University of International Business and Economics, Shanghai, China

**Keywords:** internal branding, person–organization fit, intent to stay with the brand, brand performance, nonprofit organization

## Abstract

As a value-led entity, the nonprofit depends on its staff for the delivery of the brand value outsides and thus promoting the brand inside is crucial to the development of the nonprofits. Using a sample of 290 full-time staff working in 270 nonprofits in China, two related studies were conducted. Study 1 aimed to develop and validate a new scale for internal branding in the nonprofit context, while Study 2 aimed to investigate the linking mechanism between internal branding and brand performance with the mediating roles (including chain mediation) of the person–organization fit (POF) and intent to stay with the brand (IntSB). As predicted, the results revealed that: (1) the nonprofit internal branding (NIB) scale is a three-dimensional construct that is composed of brand-centered training, internal brand communication, and brand-oriented leadership, (2) internal branding positively predicts POF, IntSB, and brand performance, and (3) POF and IntSB sequentially mediate the internal branding–brand performance relationship. The implications of our findings for internal branding in the nonprofit context are discussed.

## Introduction

Given the current environment of nonprofit organizations (NPOs), characterized by increasing competition and scarcer resources, the concept of branding has attracted significant attention in the nonprofit management literature. This concept emphasizes the brand as core equity for the organization and calls for building a strong brand for NPOs to gain a competitive edge. The success of service branding largely relies on the service providers (i.e., employees; Ayrom and Tumer, 2020). Employees are particularly important in building a strong and distinctive nonprofit brand because the nonprofit sector is labor-intensive, mainly offers intangible services. Employees of such organizations offer customers a unique brand experience that cannot be imitated by competitors (King and Grace, 2010; Yang et al., 2015). Thereby, NPOs have to include their staff in the overall brand management strategy and align them with brand values. Internal branding refers to the effort of an organization to promote and inculcate brand values to its internal stakeholders (Aurand et al., 2005), thus enabling employees to understand and identify with brand values and practice them in their daily work. As Liu et al. (2015) purported, employee-oriented internal branding is of great significance to NPOs because internal branding is not only a cost, but also an investment.

Despite the importance of internal branding for NPOs, there have been surprisingly few attempts to examine this topic in the nonprofit context. On the one hand, what constitutes the Scale of nonprofit internal branding (NIB) is still understudied. While some studies have examined the conceptual development of internal branding in the commercial context, however, employment sector heterogeneity such as organizational structure and employee characteristics (Lee and Wilkins, 2011), decreases their generalization to the NPO literature. The role of branding with employees in the whole public sector that comprises the nonprofit is still underexplored (Leijerholt et al., 2020; Barros-Arrieta and García-Cali, 2021). On the other hand, the research on the mechanism linking internal branding and brand performance has remained elusive to date. The brand performance is deemed an important indicator to assess the quality level of employee services against brand standards (Punjaisri and Wilson, 2011). There exist a growing number of studies investigating internal branding in engendering individuals’ brand-supportive attitudes and behaviors in the commercial sector (Punjaisri et al., 2009; Yang et al., 2015). However, current research regarding internal branding still lacks knowledge about the conductive pathway from internal branding to brand performance, especially in the nonprofit scene.

In this paper, we sought to expand the research on internal branding by focusing on nonprofit employees and their perceptions of internal branding determinants, and thus two related studies are conducted. In Study 1, we develop and validate the NIB scale. To the best of our knowledge, this study develops the first-ever NIB scale in the branding management literature. As such, this study introduces a new construct that helps better understand the nuances of internal branding in a nonprofit setting. In study 2, we explore the effects and mechanism of internal branding that lead to nonprofit employees’ brand performance with two potential mediators namely person–organization fit (POF) and intent to stay with the brand (IntSB), on which few study has been done. POF is conceptualized as the value congruence between people and organizations (e.g., Cable and DeRue, 2002; Kristof-Brown et al., 2005) and is thus an immediate product of the internal branding scheme. Along with the argument that value congruence could influence individuals’ brand performance (Baker et al., 2014); POF is proposed as a mediator in the internal branding–brand performance relationship. For the other mediator, IntSB describes one’s willingness to remain with the organization brand, and is a form of job retention intention that is deemed the predictor of actual turnover in the organizational behavior literature (Tett and Meyer, 1993). Based on the two mediation mechanisms proposed above, Study 2 also aims to provide a more complete picture of how internal branding influences brand performance by positing the chain mediating roles of POF and IntSB. We expect that, for NPO employees, internal branding efforts foster POF, in turn positively impacting IntSB and eventually enhancing brand performance.

## Nonprofit Internal Branding: Construct Definition and Development

### Construct Definition for Nonprofit Internal Branding

The concept of internal branding was first proposed by Berry and Parasuraman (1991) and referred to the process of explaining and selling the brand to employees. Contrary to external branding, which highlights the recognition and satisfaction of the specific needs of external stakeholder (especially customers); internal branding has employees as its focus and highlights their crucial role in delivering the brand promise and realizing brand value. Internal branding primarily aims at developing and enhancing the shared values between employees and employers (Foster et al., 2010). NPOs are mission-driven organizations and attract those who identify with the organizational values to provide services meeting stakeholders’ expectations. Hence, internal branding targets internal members and facilitates their “buy-in” of brand values and should be given priority by NPOs. The nonprofit employees are willing to invest their energy to the mission of the organization if they buy into an organization’s values and mission (Akingbola and van den Berg, 2019); besides, the clear communication of brand values to employees can further enhance their brand commitment and facilitate transforming the espoused brand values into reality (Foster et al., 2010; Liu et al., 2015). Since the concept was put forward by Berry and Parasuraman (1991), internal branding has attracted the growing attention of the academia, and different views on its conceptualization arose. Some researchers considered that internal marketing mainly facilitates employees’ brand awareness and emphasized that communication plays a vital role in employees’ internalization of brand values (Thomson et al., 1999; Berry, 2000). However, internal branding could be also deemed a process of aligning employees’ behaviors with brand values (Burmann and Zeplin, 2005; Vallaster and de Chernatony, 2005). Therefore, from a holistic perspective, internal branding should extend beyond brand communication activities to include human resource practice (e.g., training, development, and rewards; Papasolomou and Vrontis, 2006; Punjaisri and Wilson, 2011; Lee et al., 2014). Recently, Barros-Arrieta and García-Cali (2021) conducted a systematic literature review of internal branding and categorized it into three key components, namely brand-centered human resource management, brand leadership, and internal brand communication.

Although a universal conceptualization has not been proposed yet, the holistic perspective appears to be the most utilized in more research of internal branding. Besides, many scholars agreed that the essence of internal branding practices lies in enabling employees to acquire and understand organizations’ brand knowledge, which in turn facilitates the transformation of the brand promise into reality (e.g., Yang et al., 2015). For instance, Punjaisri et al. (2009) referred to internal branding as a nurturing process, whereby employees are communicated and trained in line with brand knowledge. As such, in our study, the holistic approach that we adopted emphasizes the education of brand knowledge (e.g., mission, goals, and brand performance standards) to employees when conceptualizing NIB, and thus internal branding is perceived as the practices of communicating and educating employees in terms of brand knowledge and information with the purpose of persuading them to buy into brand values. In this manner, for NPOs, internal branding comprises the activities devoted to the sharing of brand knowledge and ideas to employees and other activities not involving the communication of brand knowledge such as recruiting and rewarding.

### Construct Development for the Nonprofit Internal Branding

A through literature review that is not the main part of this paper, has been undertaken to initially identify key factors of the NIB scale. By adopting the mentioned-above holistic view and based on other theoretical backgrounds on internal branding (e.g., Berry and Parasuraman, 1991; Barros-Arrieta and García-Cali, 2021), we initially identified three factors that might constitute the NIB scale, and these three factors are: (1) brand-centered training; (2) internal brand communication; and (3) brand-oriented leadership.

First, brand-centered training is an organizational training scheme for educating employees about brand knowledge, being the premise for employees’ delivery of the brand promise. Many NPOs are small scaled, and the financial and human resource constraints they face are most likely to impact their internal branding. Thereby, being different from that of for-profit entities, brand-centered training for nonprofits shall stress the individualized consideration and the cost-saving action learning. Specially, NPOs shall design a personalized branding learning scheme for each member, which helps them to understand their own roles in relation to the brand mission and enhances their brand performance. Besides, in response to the early call for employees’ management participation in the nonprofit work setting by Cunningham (2001), learning based on specific job issues could be a favorable approach for instilling brand knowledge to NPO employees.

Second, in line with the opinions of some scholars (e.g., Liu et al., 2015; Buil et al., 2016), leaders can facilitate employees’ understanding and acceptance of organizational brand values. In the current study, brand-oriented leadership refers to leaders’ effort to instruct brand knowledge and information by words and deeds to their subordinates. According to social learning theory of Bandura (1977), the formation of individual behaviors is not only affected by one’s own experience, but also by one’s learning and imitation of others. Therefore, as the main link between employees and the organization’s brand values (Buil et al., 2016), leaders could construct a learning mechanism that boosts followers’ understanding and acceptance of the brand. Generally, NPOs are characterized by flat structures and limited hierarchy (Barnabé and Burns, 1994), which provides a basis for leaders’ interpersonal brand promotion to followers. As such, NPOs should utilize the short supervisor–subordinate power distance, and brand-oriented leadership thus emphasizes the intense interpersonal interaction and a follower-centric approach. Nonprofit leaders can influence how followers think of their job and enhance their perceptions of the work impact (Peng et al., 2020). In practice, serving as “integrating forces” in facilitating the internal branding activities, nonprofit leaders should “live” the brand by example while instructing subordinates. Besides, nonprofit leaders should prioritize the needs of followers, and treat followers individually rather than the same when coaching brand knowledge.

Third, similar to the views of internal branding in the commercial sector (e.g., Lynch and de Chernatony, 2004), the nonprofit internal brand communication is defined as integrated internal communications (formal and informal), including all verbal and nonverbal messages regarding the brand knowledge. However, it is worth mentioning that the open and direct communication shall be the highlights for the internal brand communication due to the more managerial flexibility and less bureaucracy of the nonprofits. Besides, the nonprofit individuals gathered to pursue their common values, and thus are more inclined to discuss and exchange their values or organizational values (Ihm and Baek, 2020). As such, the value-related information exchange rather than practical talks (e.g., task technical information) shall be prioritized in the NPO internal communication. Overall, brand-centered training, internal brand communication, and brand-oriented leadership can represent well the efforts that NPOs make to enhance employees’ brand knowledge.

## The Linkage Between Internal Branding and Brand Performance

### Internal Branding as a Predictor of Brand Performance

As a type of individuals’ task performance, brand performance describes the extent to which individuals deliver the brand promise of their organization (Punjaisri et al., 2009). Although prior studies have supported the positive influence of internal branding on employees’ brand performance in the commercial sector (e.g., Punjaisri and Wilson, 2011; Garas et al., 2018), this relation in the nonprofit context has so far been disregarded. In this study, internal branding is proposed as a predicator of brand performance for NPO employees. The exchange and sharing of information in the organization (e.g., brand knowledge) is generally deemed a key component of a high-performance work system (Hooff and Ridder, 2004). In practice, for NPOs, internal branding enables employees to acquire brand-related knowledge and information, such as values, mission, brand development history, and the standards of brand promise delivery, thus engendering the increase of their brand performance. As Vallaster and de Chernatony (2005) argued, it is through the internalizing the organizational brand values that employees can adhere to corresponding brand standards in their specific work tasks.

In this study, social exchange theory (Blau, 1964) is drawn on to further clarify the internal branding–brand performance relationship. The core idea of social exchange theory is the reciprocity principle that individuals have the obligation to reciprocate each other’s favorable treatment. In effect, the effort of employers to help employees to acquire and understand brand knowledge through an internal branding scheme represents employers’ favorable treatment toward employees, and thus facilitates the establishment of social exchanges between employers and employees. Reciprocity occurs in a mature social exchange relationship and an individual will reciprocate the employer’s fair treatment (Turnley et al., 2003). Task performance (e.g., brand performance) is often viewed as a type of acceptable currency with which an individual reciprocates the favorable treatment by the organization (Settoon et al., 1996). Thereby, employees are motivated to engage in working behaviors consistent with the brand promise as a result of the reciprocity principle. As Lee et al. (2014) argued, the increase of employees work input is a means of returning the organization’s treatment of them as internal customers. That is, employees feel obliged to reciprocate the employers’ favorable treatment (i.e., internal branding) and, thus, perform in such a way that they meet brand standards (i.e., brand performance).

### Internal Branding as a Predictor of Person–Organization Fit

Being related to employee-environment compatibility, person-organization fit (POF) describes “the compatibility between people and organizations that occurs when: (a) at least one entity provides what the other needs, (b) they share similar fundamental characteristics, or (c) both” (Kristof, 1996, p. 4–5), and is divided into supplementary fit and complementary fit. Generally, the match between individuals’ personal characteristics and job attributes is treated as complementary fit, while an individual’s match with the values, goals, and mission of the organization as a whole is often treated as supplementary fit (Edwards, 1991; Kristof-Brown et al., 2005). To date, little attention has been paid to the internal branding–POF relationship. In this study, we propose that internal branding enhances POF for nonprofit employees.

Internal branding is a useful tool that enables employees to understand their organization’s brand values and expectation more accurately and convinces them of the brand’s relevance and worth (Thomson et al., 1999). It thus represents an important mechanism whereby employees receive brand-related information of the organization. For nonprofit employees, internal branding contributes to forming a deeper and clearer understanding of brand values and engenders the “buy-in” of brand values. As a result, the value congruence between individuals and the organization will increase. In other words, internal branding plays an important role in increasing employees’ POF. Some previous studies have also shown that human resources management practices (e.g., train and development) could help to align employees with the organizations’ values (e.g., Kooij and Boon, 2018; Luu, 2018). Moreover, social exchange theory (Blau, 1964) helps explain the internal branding–POF relationship. As previously mentioned, an internal branding strategy is viewed as employers’ favorable treatment towards employees. As the organization helps employees to understand the brand values of the organization, an internal branding strategy represents employers treating employees as internal customers. Consistent with social exchange theory, employees who enjoy organization’s favorable treatments (e.g., brand training and brand leadership) develop a sense of being valued by the organization and, in return, feel obliged to reciprocate with positive work outcomes (e.g., affirming to the values and norms of the organization) and exhibit high compatibility with organization (i.e., POF).

### Internal Branding as a Predictor of Intent to Stay With the Brand

Intent to stay with the brand (IntSB) refers to an individual’s intention to remain with the organizational brand on a long-term basis and represents employees’ support for the organizational brand through continued organization membership. Internal branding fosters the positive perceptual exchange between employees and employers (Ayrom and Tumer, 2020). For the nonprofit employees, internal branding helps them internalize organizational brand values, leading a link between themselves and the brand, and understand the connection between their personal growth goals and the organizational mission, all of which engender their sense of ownership and encourage them to remain with the organization in the long term. While one recent study by Dechawatanapaisal (2018) demonstrated that internal branding predicts employee retention, the internal branding—IntSB relationship is still elusive. In this study, we employ organizational socialization theory (Schein, 1968; Fisher, 1986) to further clarify this relationship.

Organizational socialization refers to the process through which new members turn from “outsiders” to “insiders” by gaining knowhow on the organization (Schein, 1968) and impacts the adjustment of individuals (especially newcomers) to their jobs, groups, and organizations (Fisher, 1986; Ashforth et al., 2007). Through organizational socialization, individuals (both new and existing staff) turn into proficient and comfortable members of the organization. According to the theory of organizational socialization, when experiencing high uncertainty in the workplace, individuals often have difficulties in meeting job requirements, thus engendering low job satisfaction and high turnover intention. Internal branding servers to make the workplace more desirable for staff (Ayrom and Tumer, 2020). In practice, an internal branding strategy could thus help resolve the issue of job uncertainty for employees, as it enables them to acquire and understand the required brand-related knowledge and skills for performing the job. They also benefit from the strong social support brought by the internal branding scheme (e.g., peers’ communication and leaders’ induction), which helps them establish a predictable work environment at multiple levels (e.g., work requirements, organizational norms and culture, and interpersonal interactions) and reduce uncertainties in the workplace. Moreover, as the process of educating employees in terms of brand knowledge, internal branding results in employees’ good mastery and recognition of organizational brand’s knowledge (e.g., brand value), which is important for employees’ organizational socialization. As a result, internal branding facilitates employees’ organizational socialization process, characterized by better adjustment to the job and transference from outsiders into insiders; as such, it strengthens employees’ bonds with the organizational brand and, eventually, the tendency of maintaining the organization membership and serving the organizational brand.

### POF as a Mediator Between Internal Branding and Brand Performance

Value acts as a guide for behaviors of an individual in any given setting, and this case might occur more often in the nonprofits of which the shared values are at the core (Akingbola and van den Berg, 2019). In other words, nonprofit employees with high POF share values and goals with the organization and are expected to have a clear understanding of organizational expectations, thus tending to exhibit desirable behaviors for the organization (e.g., brand performance). While some prior studies have unveiled that the fit between an individual’s personal values and those of the organization positively relates to his or her task performance (e.g., Hamstra et al., 2019), the POF–brand performance relationship has yet to be examined. In this study, this relationship is further explained from the viewpoint of cognition. The person–organization lack of fit involves an experience of cognitive dissonance that could evoke role conflict (Tong et al., 2015). Individuals low on POF are likely to experience a trade-off between what they have to do and what they wish to do (Maslach and Leiter, 2008) and, thus, tend to put less effort in the workplace (Kilroy et al., 2017). For instance, employees’ misfit with the norms and values of the organization would result in a decrease in job satisfaction (Lim et al., 2019), which is an important predicator of job involvement and performance (Wegge et al., 2007; Wright et al., 2007). Conversely, employees high on POF are inclined to define themselves in terms of their organizations (Saks and Ashforth, 1997). They thus experience little cognitive dissonance and hold a favorable attitude toward the organization, which motivates them to be more engaged in delivering the brand promise (brand performance). In short, POF will be positively related to brand performance. As mentioned before, POF might be the outcome of internal branding for nonprofit employees. Therefore, we propose that POF is the underlying mechanism through which internal branding enhance nonprofit employees’ brand performance.

### IntSB as a Mediator Between Internal Branding and Brand Performance

An individual’s attitude toward the brand could influence the manner in which he or she delivers a service. Prior studies have demonstrated that positive brand attitudes (e.g., brand commitment and brand identification) lead to an increase in employees’ brand-supportive behaviors (e.g., Punjaisri et al., 2009; Yang et al., 2015). Similarly, as a form of brand-supportive attitude, IntSB may be the potential predicator of brand performance. Generally, the main purpose of an individual working hard and showing high-quality work performance is to gain employer’s recognition and positive returns (e.g., job promotion, high salary and welfare). As such, employees with low IntSB are unlikely to devote more effort to accomplishing the missions and goals of the organization, thus decreasing their work input and showing low self-discipline in the workplace. That is, they would be unwilling to fulfill the promise of the organization brand to external constituents if they plan to leave the organization in the future. Conversely, for employees with high IntSB, the goal of acquiring the recognition and returns of the organization will trigger them to set a high standard of work performance and show more brand-supportive behaviors (e.g., brand performance). Therefore, IntSB positively predicts brand performance for nonprofit employees. In combination with the mentioned above proposal that internal branding will enhance employees’ IntSB, internal branding will affect brand performance through IntSB.

### The Chain Mediating Role of POF and IntSB Between Internal Branding and Brand Performance

The similarity attraction paradigm underlying value congruence indicates an individual is more attracted to and trusting of others with whom he or she shares similar characteristics (Cable and Edwards, 2004). For instance, a high POF shows the establishment of an individual–organization relationship characterized by improved communication and high trust levels (Edwards and Cable, 2009). As such, POF, typically conceptualized as value congruence, could be conducive to strengthening the membership of an individual with the organization. As Cable and DeRue (2002) argued, POF helps develop bonds between an individual and an organization. The value congruence between an individual and an organization may influence the individual’s perception of need fulfillment and contribute to the development of a favorable attitude toward the organization, which will increase employees’ intent to remain with organization (Boon and Biron, 2016; Newton and Mazur, 2016). Moreover, the nonprofit management literature asserted that charisma and mission identification are valuable tools for attracting and retaining nonprofit employees (Ihm and Baek, 2020; Peng et al., 2020). Thus, POF enhances IntSB, which is a key premise for the establishment of chain mediation in the current study. Despite these findings, there are still no studies that investigate the chain mediating role of POF and IntSB. Therefore, we propose that POF and IntSB sequentially mediate the effect of internal branding on brand performance for nonprofit employees.

## Study 1

Study 1 aimed to develop an instrument to assess NIB with employees. In line with our conceptualization of internal branding in the nonprofit context, we expect the NIB measurement has three factors: (a) brand-centered training; (b) internal brand communication; and (c) brand-oriented leadership. Thus, we expect:

*H1*: The NIB scale is a three-dimensional construct that comprises brand-centered training, internal brand communication, and brand-oriented leadership.

### Methodology

#### Participants and Procedure

The data were collected from the nonprofit sector in China. With the assistance of local governments, we used convenience sampling and administered on-site (paper-and-pencil) and online surveys to 380 full-time staff from 270 NPOs in several cities (e.g., Beijing, Shanghai, and Changsha) in China. An informative letter that emphasized the confidentiality and anonymity of responses was also distributed along with the questionnaires and written informed consent was obtained from all participants. Finally, 290 valid questionnaires were returned. Among the respondents, 38.6% were male and 72.4% were married; the age of the respondents is mainly in the 26–35 years old group (40.3%), followed by those in the 36–45 years old group (26.6%); in terms of education, the majority of respondents held a bachelor’s degree (57.2%), followed by those with a vocational education program (27.6%); the average tenure was mainly 3–5 years (43.4%) or 6–10 years (26.9%).

Following Walumbwa et al. (2008) and Zuberbühler et al. (2021), two procedures were used in developing and validating the scale measuring NIB: (a) construct development; (b) item development and validation. In the procedure of construct development, based on an extensive literature view of internal branding, the construct definition was presented, and content specifications of the NIB construct were initially identified as being composed of three distinct but related substantive factors: brand-centered training, internal brand communication, and brand-oriented leadership.

In line with the suggestions by Churchill (1979), the procedure of item development and validation included three steps: (1) initial item generation; (2) item refinement; and (3) validity assessment. In Step 1, the initial items were generated through combined deductive and inductive approaches (Hinkin, 1995). First, the initial item generation began with focus group interviews. The interviews involved 35 fulltime employees (including supervisors and subordinates) from nine NPOs covering three typical organizational forms in China, namely member-based societies (e.g., academic societies), philanthropic foundations (e.g., education foundations), and social service agencies (e.g., nursing homes). During the interviews, participants were invited to describe the strategies that employers use to disseminate brand knowledge to employees with examples and freely express their views on these strategies. Further, using an open-coding approach (Miles and Huberman, 1994), content analysis was conducted to generate the initial items of the NIB scale. Second, we reviewed the relevant literature and critically examined the established instruments for evaluating internal branding (e.g., Punjaisri and Wilson, 2011; Piha and Avlonitis, 2018), which helped to improve the construct validity of the scale. Through these two approaches, an initial list of 24 items was generated with three themes representing the NIB dimensions, namely brand-centered training, internal brand communication, and brand-oriented leadership.

In Step 2, three scholars in the NPO management filed were independently invited to examine the initial item list. In this step, the theoretical rationale was critically scrutinized and the construct items were carefully checked, after which the experts rated the degree to which an item matched the construct definition in the current study. Finally, the 18 items that match the related dimensions of NIB scale were retained and the revised questionnaire was also provided to three university professors and five nonprofit practitioners to check items’ phrasing for clarity and readability. To further refine the items and examine the proposed multidimensionality, a quantitative approach called exploratory factor analysis (EFA) was adopted. In Step 3, confirmatory factor analysis (CFA) was used for the final verification of the proposed scale, and the construct model was assessed from both convergence validity and discriminant validity. Generally, two independent groups of samples are required for the cross-validation of a new scale (Anderson and Gerbing, 1988). The observation number should be over five times the item number of the original scale in the EFA (Gorsuch, 1983) and over 10 times the item number of the original scale in the CFA (Thompson, 2000). Therefore, the 290 valid observations were divided into two subsamples at random, that is, sub-sample 1 (*n* = 110) was used for EFA and sub-sample 2 (*n* = 180) for CFA.

### Results

The factor structure of the NIB scale was tested with EFA followed by CFA in IBM SPSS version 26.0. The EFA was conducted with subsample 1 as follows. First, the Bartlett test of sphericity was used to verify the appropriateness of factor analysis and the Kaiser-Meyer-Olkin (KMO) test to judge the sampling adequacy (*N* = 110, in this case). Generally, if the KMO value is more than 0.60 and significant, the data are suitable for factor analysis (Hair et al., 2010). For the 18-item NIB scale (*N* = 110), the KMO value was 0.924 and the Bartlett test of sphericity was significant (*p* < 0.01), thus indicating the suitability of factor analysis. Second, EFA was conducted using principal component analysis with varimax rotation and Kaiser normalization (factors with eigenvalues > 1). The initial results revealed a three-factor solution accounting for 71.67% of the variation in the data. Three items with cross-loadings above 0.4 were removed from the list. Therefore, the new scale included 15 items (see Table 1) and we then conducted another round of principal component analysis. For the new three-factor solution (shown in Table 1), the factor loadings for all items were above 0.50 by Gorsuch (1997), ranging between 0.631 and 0.879, with no cross-loading items. Moreover, the new three-factor solution accounted for 74.79% of the variation in the data, which was nearly 3% higher than the original one, indicating the effectiveness of the item reduction. Factor 1, “brand learning action,” assesses the extent to which an individual perceives employer’s efforts to educate him or her to brand knowledge through training and practice activities. Factor 2, “internal brand communication” assesses the degree of an individual’s perceived feeling of brand knowledge sharing within the organization. Factor 3, “brand-oriented leadership,” assesses the degree of an individual’s perceived feeling of being instructed and guided by his or her supervisors on brand knowledge and information.

**Table 1:** A principal component analysis of the 15-item nonprofit internal branding (NIB) scale.

Dimensions and items	Component		
	1	2	3
**Brand-centered training**			
1. Training gives me appropriate skills for delivering the brand promise based on brand standards	0.796	0.226	0.367
2. Training introduces to me the brand values and brand mission	0.879	0.127	0.264
3. Training, especially action learning opportunities, gives me essential information about the brand, so that I can show work behaviors that are consistent with the brand promise	0.866	0.289	0.105
4. I clearly understand my role in relation to the brand mission, through the personalized training scheme designed and provided by my organization	0.843	0.269	0.246
5. The organization provides me with flexible, diverse training activities (e.g., case-study/buddy/on-the-job training) so that I can perform the work satisfying brand expectations	0.656	0.300	0.365
**Internal brand communication**			
1. The organization regularly holds meetings for brand-related communication and discussion where I have a voice	0.312	0.262	0.765
2. The organization utilizes various ways (e.g., brochure and WeChat) to communicate brand information, especially brand values, to me	0.209	0.132	0.834
3. There are frequent between-department and between-individual communications concerning the brand (e.g., mission and core values) in the organization	0.311	0.289	0.722
4. There are open and direct communication channels for brand information within the organization	0.222	0.357	0.631
5. Internal communication enables me to know the marketing performance of the brand at all times	0.164	0.280	0.716
**Brand-oriented leadership**			
1. My supervisor plays an exemplary role in delivering high-quality services that meet the brand’s standards	0.262	0.812	0.205
2. My supervisor well performs his or her job according to brand expectations	0.152	0.809	0.212
3. My supervisor cares whether and how I, as an individual, understand the brand values	0.194	0.788	0.344
4. My supervisor often shares with me his/her understanding of the brand and coaches me as an individual	0.291	0.828	0.204
5. My supervisor regularly talks to me about the most important brand information (e.g., mission and brand standards)	0.247	0.742	0.328

*n* = 110.

#### CFA and Validity Analysis

Using subsample 2, a series of CFAs was carried out to validate the proposed model, comprised of three latent variables. To examine the distinctiveness of the three dimensions of the NIB construct, we compared our baseline model (i.e., three-factor model) against a series of alternative nested models, merging two or three of the NIB dimensions. The root-mean-square error of approximation (RMSEA), comparative fit index (CFI), and values for the incremental fit index (IFI) were used to assess the model fit according to Bentler (1990) (i.e., RMSEA > 0.08, CFI > 0.90, IFI > 0.90).

Following Johnson et al. (2011, 2012), we examined the multi-dimensional construct by performing first- and second-order CFAs and used three criteria: (a) the indicator variables should significantly and substantially load on the second-order factor (factor loading > 0.70); (b) the second-order model should yield a good fit; and (c) the set of indicators should have high internal consistency. As per Table 2, compared with three alternative two-factor first-order models and one-factor model, the proposed three-factor models (first- or second-order) yielded a better fit with the data (*x*^2^/df = 1.779, df = 87; RMSEA = 0.07, CFI = 0.97, and IFI = 0.97). However, for the three-factor first-order model, the correlation coefficients among the three subscales of the NIB scale were medium and high (>0.50), which implies a possible common higher-order factor (i.e., internal branding). According to Bollen (1989), a second-order model is preferable to a first-order one if it fits the data because it allows for covariation among first-order factors and accounts for the corrected errors common in first-order models. As such, we further examined the validity of the three-factor second-order model. The results showed that 15 items loaded significantly on the corresponding latent variables, with the standard factor loading ranging between 0.68 and 0.86, and three subscales loaded significantly and substantially on the higher second-order factor NIB Scale (standard factor loading: 0.910 for brand-centered training; 0.848 for internal brand communication; and 0.872 for brand-oriented leadership).

**Table 2:** Measurement model comparisons.

Model	*df*	χ^2^	χ^2^/*df*	CFI	IFI	RMSEA
Proposed three-factor, first-order model	87	154.735	1.779	0.97	0.97	0.07
Proposed three-factor, second-order model	87	154.735	1.779	0.97	0.97	0.07
Alternative two-factor, first-order model						
Merging a and b	89	337.539	3.793	0.90	0.90	0.13
Merging a and c	89	401.204	4.508	0.87	0.87	0.14
Merging b and c	89	364.38	4.094	0.88	0.89	0.13
Alternative one factor model: Merging a, b, and c	90	557.773	6.197	0.81	0.81	0.17

*n* = 180; a, brand-centered training; b, internal brand communication; and c, brand-oriented leadership.

Moreover, the average variance extracted (AVE) was used to assess construct validity. According to Fornell and Larcker (1981), the AVE for each NIB dimension should be above 0.50 (assessing convergent validity) and more than the square of the correlation (*R*^2^) between it and any other dimension (assessing discriminant validity). For the three-factor second-order model, the AVE for each dimension was satisfactory (0.786 for brand-centered training; 0.707 for internal brand communication; and 0.807 for brand-oriented leadership). The *R*^2^ value for the correlation between any two NIB dimensions ranged from 0.484 to 0.570, being obviously lower than the related AVE value. Further, the AVE accounted for in the second-order factor (i.e., internal branding) by its three first-order factors was 0.86, being above threshold of Fornell and Larcker (1981). Therefore, there exists strong evidence of convergent and discriminant validity for the three-factor second-order model. As a result, for the proposed three-factor models, the parsimonious second-order model could be more desirable, as it was consistent with the theoretical constructs of NIB: *brand-centered training, internal brand communication*, and *brand-oriented leadership*. Thus, H1 was supported.

### Brief Discussion of Study 1

In line with our conceptualization of internal branding for NPOs, the results of Study 1 confirmed the three-dimensional NIB construct. More specifically, the factor structure of NIB comprised three independent but positively correlated factors, namely brand-centered training, internal brand communication, and brand-oriented leadership. Besides, for the three-factor construct reflecting internal branding in the nonprofit context, a second-order factor model was confirmed with good convergent and discriminant validity.

## Study 2

This study is to exam if and how internal branding is associated with nonprofit employees’ brand performance (*via* POF or/and IntSB). The hypothesized model was explored through the hypotheses as following:

*H2*: Internal branding is positively related to employees’ brand performance.

*H3*: Internal branding is positively related to employees’ POF.

*H4*: Internal branding is positively related to employees’ IntSB.

*H5*: POF plays a mediating role in the internal branding–brand performance relationship.

*H6*: IntSB plays a mediating role in the internal branding–brand performance relationship.

*H7*: POF and IntSB sequentially mediate the internal branding–brand performance relationship.

**Figure 1:**
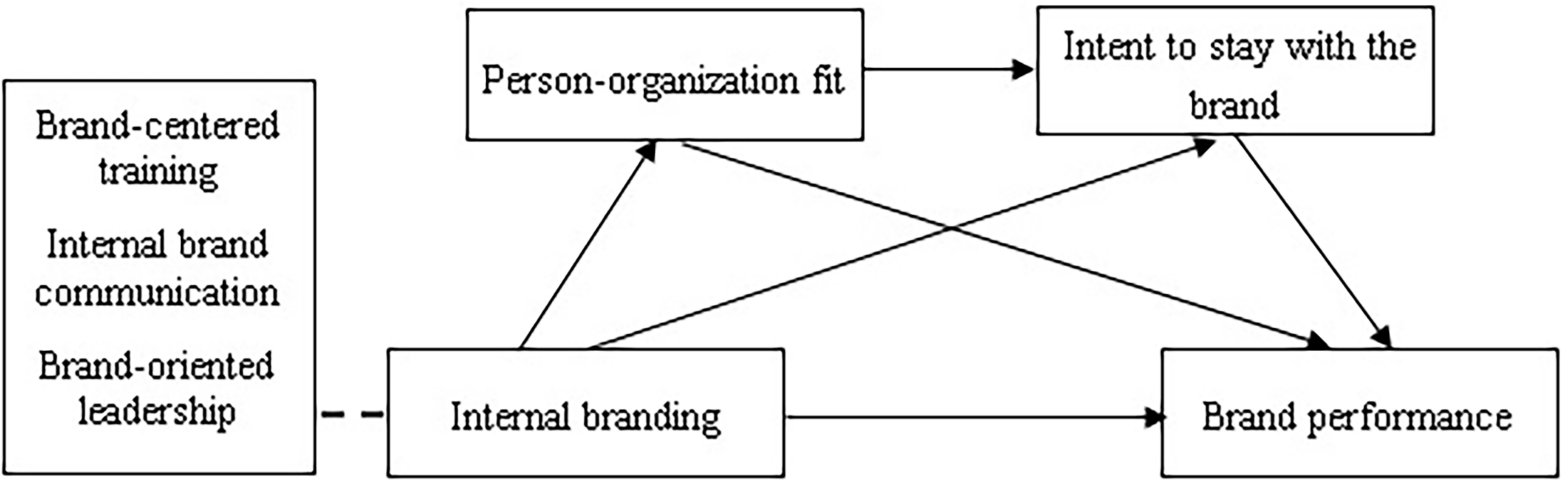
The conceptual model.

The conceptual framework of Study 2 is presented in Figure 1.

### Methodology

#### Participants and Procedure

To increase the efficiency of data collection for the manuscript, the questionnaires distributed to participants in Study 1 included the items of the measures for Study 2, and the elimination of the participants with missing data for Study 2 was considered in the process of validating the questionnaires for Study 1. Thereby, the sample of Study 2 is the same as that of Study 1 with 290 participants in the nonprofit sector.

#### Measurement

In Study 2, internal branding is measured on a five-point Likert response scale ranging from 1 (strongly disagree) to 5 (strongly agree), while POF, IntSB, and brand performance on a seven-point Likert response scale ranging from 1 (strongly disagree) to 7 (strongly agree).

##### Internal Branding

The scale is a new measurement that Study 1 has developed, designed to assess NIB. The NIB scale is a 15-item construct that combines three dimensions: brand-centered training (five items); internal brand communication (five items); and brand-oriented leadership (five items). The scale’s reliability is 0.955.

##### Person–Organization Fit

Person–organization fit (POF) was measured by the four-item scale developed by Saks and Ashforth (1997). One sample item is “The values of the organization are similar to my personal values.” The scale’s reliability is 0.936.

##### Intent to Stay With the Brand

The four items of the IntSB measurement are mainly revised from the prior scales (Chen, 2001; Hu et al., 2018). One example item is “I will stay with the organization brand for quite a while.” This scale’s reliability is 0.885.

##### Brand Performance

Brand performance was measured by the four-item scale developed by Punjaisri et al. (2009). One sample item is “The quality level of my services meets the brand standards of the organization.” The scale’s reliability is 0.917.

##### Control Variables

Age, gender, marital status, education level, tenure, and position level were controlled for due to their potential effects on the dependent variables of the study.

### Results

#### Descriptive Statistics and Correlations

Table 3 shows the descriptive statistics and inter-correlations for all variables. As expected, all bivariate correlations for the variables in the hypotheses were statistically correlated in the anticipated directions. For instance, internal branding correlated positively with POF, IntSB, and brand performance.

**Table 3:** Descriptive statistics and correlations.

S. no		1	2	3	4	5	6	7	8	9	10
1	Gender	1									
2	Age	0.056	1								
3	Education	0.034	0.117^*^	1							
4	Marriage	0.017	0.546^**^	0.047	1						
5	Position level	0.122^*^	0.247^**^	0.089	0.263^**^	1					
6	Tenure	0.066	0.365^**^	0.030	0.434^**^	0.259^**^	1				
7	Internal branding	0.003	0.005	0.086	0.025	0.042	0.040				
8	POF	0.029	0.067	0.055	0.054	0.018	0.078	0.666^**^	1		
9	IntSB	0.003	0.073	0.051	0.043	0.026	0.023	0.548^**^	0.568^**^	1	
10	Brand performance	0.038	0.045	0.044	0.066	0.034	0.085	0.526^**^	0.516^**^	0.504^**^	1

*n* = 290.

* *p* < 0.05 and

** *p* < 0.01.

#### Confirmatory Factor Analysis

All examined variables were self-reported in this study. To detect and control the influence of common method bias and examine the discriminant validity of the variables, a series of CFAs (i.e., four-, three-, and two-factor models) were conducted. In comparison to the three-factor model (IntSB and brand performance are combined; χ^2^/df = 3.198; RMSEA = 0.087; IFI = 0.897; CFI = 0.896, and TLI = 0.886) and two-factor model (POF, IntSB, and brand performance are combined; *x*^2^/df = 4.103; RMSEA = 0.104; IFI = 0.854; CFI = 0.853, and TLI = 0.839), the four-factor model yielded a better fit (*x*^2^/df = 1.812; RMSEA = 0.053; IFI = 0.962; CFI = 0.962, and TLI = 0.958) and is above the mentioned-above criterion (Bentler, 1990), suggesting good discriminant validity.

#### Testing Direct and Mediation Effects

To test and validate internal branding’s relationships with POF, IntSB and brand performance, we established three mediation models (see Table 4) and used bootstrapping procedures with the aid of process of Hayes (2013). Model 1 and Model 2 aimed to test the mediation role of POF and IntSB respectively, while Model 3 aimed to text the chain mediation role of POF and IntSB. As shown in Table 4, the direct effects of internal branding on both POF and IntSB were significant (POF: *b* = 0.393, 95% CI [0.286, 0.500], see Path 1a; IntSB: *b* = 0.353, 95% CI [0.243, 0.463], see Path 2a). Thus, Hypothesis 3 and Hypothesis 4 were support. Besides, Table 4 shows, the indirect effect of internal branding on brand performance through POF was significant (*b* = 0.156, 95% CI [0.098, 0.235]; see Path 1c). Given that the direct path from internal branding to brand performance was significant, it can be concluded that POF played a partial mediating role between internal branding and brand performance. Hence, Hypothesis 5 was supported. Similarly, the results (see Table 4) show that the indirect effect of internal branding on brand performance through IntSB was significant (*b* = 0.139, 95% CI [0.084, 0.213]; see Path 2c), while the direct effect of internal branding on IntSB was significant. We can conclude that IntSB played a partial mediation role between internal branding and brand performance. Hence, Hypothesis 6 was supported.

**Table 4:** Direct, indirect, and total indirect effects for the model.

Path	Effect	Boot SE	BootLLCI	BootULCI
**Model 1: Internal branding–POF–brand performance**				
**Direct effect**				
Path 1a: internal branding → POF	0.393	0.054	0.286	0.500
Path 1b: internal branding → brand performance	0.274	0.053	0.170	0.378
**Indirect effect**				
Path 1c: internal branding → POF → brand performance	0.156	0.035	0.098	0.235
**Model 2: Internal branding–IntSB–brand performance**				
**Direct effect**				
Path 2a: internal branding → IntSB	0.353	0.056	0.243	0.463
Path 2b: internal branding → brand performance	0.292	0.052	0.189	0.394
**Indirect effect**				
Path 2c: internal branding → IntSB → brand performance	0.139	0.033	0.084	0.213
**Model 3: Internal branding–POF–IntSB–brand performance**				
**Direct effect**				
Path 3a: Internal branding → brand performance	0.233	0.052	0.131	0.335
**Indirect effect**				
Path 3b: internal branding → POF → brand performance	0.103	0.034	0.043	0.177
Path 3c: internal branding → POF → IntSB → brand performance	0.053	0.017	0.026	0.094
Path 3d: internal branding → IntSB → brand performance	0.041	0.018	0.012	0.085
**Total indirect effect**	0.197	0.039	0.131	0.284

*N* = 290; bootstrap sample size = 5,000.

Hypothesis 7 predicted that the relationship between internal branding and brand performance was sequentially mediated by POF and IntSB. As Table 4 shows, the indirect effect (internal branding → POF → IntSB → brand performance) was significant (*b* = 0.053, 95% CI [0.026, 0.094]; see Path 3c), and the direct effect of internal branding on brand performance was significant (*b* = 0.233, 95% CI [0.131, 0.335]; see Path 3a). Hence, Hypothesis 2 and Hypothesis 7 were supported.

### Brief Discussion of Study 2

Results from Study 2 supported H2, H3, and H4, indicating that internal branding positively affects nonprofit employees’ brand performance, POF and intSB. Moreover, two hypotheses, namely H5 and H6, were partially supported, indicating a partial mediating role of both POF and intSB in the link between internal branding and brand performance for nonprofit employees. Besides, H7 was supported, suggesting a chain mediating role of POF and intSB from internal branding to brand performance. Specifically, internal branding first fosters the fit between employees and organizations, which has in turn positively influences employees’ willingness to remain with the brand, and this positive intention positively influences employees’ brand performance.

## General Discussion

Aimed at addressing the gap in internal branding research, the focus of the current study was 2-fold: first, to develop a measure of internal branding for NPOs; and second, to analyze the relationship and influencing mechanisms between internal branding and brand performance for nonprofit employees. This exploratory research has portrayed some interesting results. In the case of Study 1, the results of the EFA and CFA analyses indicated that the factor structure of the NIB scale was satisfactorily illustrated by a three-factor solution that included three independent but positively correlated dimensions (i.e., brand-centered training, internal brand communication, and brand-oriented leadership). Of these three dimensions, brand-centered training had the highest standard factor loading of 0.910, while internal brand communication had the lowest standard factor loading of 0.848 and brand-oriented leadership lied in between with a value of 0.872. Thereby, for nonprofit employees, the relative importance of internal branding dimension could be perceived as: first, brand-centered training followed by brand-oriented leadership, and lastly, internal brand communication. The empirical testing of Study 1 also confirms that all three dimensions collectively define NIB as a second-order model that consists of a second-order internal branding factor and the three first-order factors mentioned above. This implies that in the perceptions of nonprofit employees toward their employer’s implementation of internal branding practices, the three dimensions shall be complemented mutually and none can be dispensed with.

Study 2 sought to explore the important yet neglected issue of how internal branding is associated with employees’ brand performance in the nonprofit context. Firstly, the authors found that internal branding is positively related to nonprofit employees’ brand performance, which is in the line with some previous research concerning the effects of internal branding on employees’ brand performance in the commercial sector (e.g., Punjaisri and Wilson, 2011; Garas et al., 2018). This means that the implementation of internal branding practices can enhance nonprofit employees’ brand performance. Secondly, the authors also confirmed third and fourth hypotheses, and internal branding has been identified as a direct antecedent of employees’ POF and IntSB in the nonprofit context. The results are similar to the two recent studies that dealt with the direct and positive effects of internal branding on brand value congruence and employee retention, respectively (Dechawatanapaisal, 2018; Hu et al., 2018). As such, it is important to emphasize the positive effects that internal branding activities can bring to the organization and its employees. That is, promoting brand values within the organization will be more likely to achieve an increase of POF and IntSB of employees. Thirdly, in Study 2, POF has been employed as a mediator linking internal branding and brand performance, since the literature suggested that POF is an important predictor of employees’ in-role performance (e.g., Hamstra et al., 2019). As expected, we found that POF mediated this relationship, and has positive and significant effect. According to the results, internal branding will increase POF, and, through POF, it will positively influence employees’ brand performance. On the question of IntSB as a mediator, our study found that internal branding leads to brand performance through IntSB. The results indicated that, for nonprofit employees, the internal branding activities contribute to creating a feeling of match with the organization, which in turn contributes to fostering favorable behaviors toward the brand (i.e., brand performance). Lastly, Study 2 examined a new influencing mechanism of internal branding on brand performance through the chain mediating role of POF and IntSB. In line with our expectations, the study supported the chain mediating mechanism of internal branding → POF → IntSB → brand performance. It is indicating that POF and IntSB were key elements in linking internal branding to brand performance for nonprofit employees.

### Theoretical Implications

The results of the current study contribute to the literature in at least three ways. First, they contribute to the evolution of the internal branding construct by developing a three-dimensional scale for NPOs. To date, no study has concentrated on the NIB scale. The NIB scale developed in this study is thus important for the internal branding construct because the heterogeneity of the sector means a low generalizability of the internal branding conceptualization in prior studies to the nonprofit context. From a holistic perspective, we developed a conceptual model composed of three dimensions, which stresses the nonprofit-matched features (e.g., individualized consideration, subjective initiative, and tense leader-follower interaction), thus provides a better understanding of the internal branding scheme for NPOs. Moreover, similar to Hu et al. (2018), the existence of the second-order three factor for the construct confirmed that the NIB construct should not be viewed as a single construct or two-factor construct as in prior studies on the commercial sector largely focused on. This new construct may encourage the research on internal branding in the nonprofit work setting and its relationship with other dependent variables (e.g., job engagement and contextual performance).

Second, this study extends the knowledge on internal branding by demonstrating its crucial role in influencing employees’ attitudes and behaviors in the nonprofit context, as limited empirical work has been undertaken in the NPO internal branding literature. In line with prior studies on the commercial sector (e.g., Garas et al., 2018), this study highlights that internal branding is a strong predictor of brand performance for the nonprofit employees. Accordingly, this research result contributes to the social exchange theory (Blau, 1964), suggesting that internal branding practices are perceived by employees as a favorable treatment that employees will reciprocate the organization by engaging in working behaviors consistent with the brand promise. Additionally, the results indicate that internal branding positively related to POF and IntSB for the nonprofit employees. To date, there is a lack of empirical studies examining the effects of internal branding on these two variables. Particularly, the internal branding literature has rarely focused on the employee retention construct (Dechawatanapaisal, 2018). We found that internal branding is an important predicator of both POF and IntSB in the nonprofit context. Therefore, the efforts of NPOs to sell the brand inside the organization are of significance to improving employees’ value fit with the organization, as well as their intention of working for the brand. This is particularly important in the nonprofit context, which is strongly value-led and where employee retention is a challenge.

Third, this study enriches the understanding of the mediating mechanism from internal branding to brand performance by incorporating both POF and IntSB in the model. As opposed to finding of Hu et al. (2018) that value congruence fails to mediate the internal branding–brand performance relationship; our results indicated that internal branding positively relates to POF, which in turn positively relates to brand performance. Moreover, the study also provided direct insights into the mediation of IntSB for the internal branding–brand performance relationship, which has rarely been explored in the internal branding literature. As expected, we confirmed IntSB to be linking internal branding with brand performance. More importantly, this study is among the first to provide empirical evidence on the chain-mediation effects of POF and IntSB from internal branding to brand performance. In the branding literature, a recent study by Leijerholt et al. (2020) has examined the mediating role of value congruence for the effects of internal branding on employees’ brand attitudes. However, this study has not provided a complete picture of internal branding’s effects on employees due to the loss of behavioral outcomes (e.g., brand performance) in the model. Our study goes a step further by combining internal branding, POF, IntSB, and brand performance into a complete model. Specifically, this model incorporated POF and IntSB as two mediating mediators and revealed that internal branding influences brand performance sequentially through POF and IntSB in the nonprofit context. Therefore, we provide a more comprehensive account of the trickle-know positive effect of internal branding on brand performance.

### Managerial Implications

First, our findings highlight the importance for NPOs of coordinating brand-centered training, internal brand communication, and brand-oriented leadership in any NIB scheme. For management, branding learning should be an important element of the internal branding scheme. NPOs have to develop systemic training programs that include both off-the-job and on-the-job training to facilitate employees’ “buy-in” of brand values. Particularly, due to resource constraints, NPOs should encourage a reasonable action-learning mechanism to facilitate employees’ acquirement and understanding of brand knowledge. This study also reveals that communication comprises another important dimension of the NIB construct. NPOs should adopt various internal communication tools (e.g., morning brief, group meetings, organization newsletter/brochure, short video of brand story, and WeChat) and prioritize the value-related communication. Third, brand-oriented leadership is a distinct component of NIB due to the less hierarchy of the organization. Leaders should not only live the brand by exhibiting brand-supportive work attitudes and behaviors (e.g., self-discipline and high-quality service delivery), but also give the followers individualized teaching and coaching, that contribute to the building of a vivid social learning mechanism through which employees can acquire and understand related brand knowledge and their personalized role related to the brand mission.

Second, in view of the positive impacts of internal branding on employees’ POF, IntSB, and brand performance, the management should be aware of internal branding’s benefits to the organization. As a mission-driven organization, the nonprofit needs employees that match its characteristics (Hansmann, 1980) and rely on them to deliver the services that stakeholders expect, thus establishing the significance of POF and brand performance for the success of NPOs. Moreover, the global economic recession, as well as the entry of for-profit organizations in public service provision, have brought development uncertainties and thus highlighted the issue of talent turnover in the nonprofit sector. The current study indicates that internal branding is an important predicator of employees’ POF, IntSB, and brand performance, and the management can interpret these results to justify expenditure on internal branding. They also have to consider internal branding from the perspective of organizational development strategy. In other words, for NPOs, internal branding should be employed as an important management tool to enhance employees’ POF, IntSB, and brand performance, while special financial and human resources should be allocated for the development and facilitation of an internal branding strategy within the organization.

Third, there exists a chain-mediation role of POF and IntSB between internal branding and brand performance, suggesting that fostering employees’ fit with brand values as well as their intention to remain with the brand should be the focus of management. However, the management should be aware that organizations can enhance employees’ IntSB and brand performance through careful selection in terms of POF. As a mission-led entity, the nonprofit shall attach great importance to the highly valued aspects of an applicant when hiring staff (Newton and Mazur, 2016). The individual values are difficult to change and employers should not overlook the issue of recruiting the “right” candidates whose values match with the organization (Foster et al., 2010). In practice, psychological tests or assessments could be employed in the selection procedures to select and recruit individuals with high POF that could improve the likelihood of employees’ IntSB and brand performance. Further, IntSB’s mediation effect also indicates that organizations should care more about the employees with low intention to stay with the brand. For instance, the management could establish a monitoring and assessment system of employees’ IntSB by scientifically analyzing reasons and trends of employee turnover, as well as identifying those who show signs of leaving the organization.

### Limitations and Future Study Directions

The present study is not without limitations. First, given that the sample was composed of employees in the nonprofit sector in China, the results might not be generalizable to other sectors and contexts. To improve the generalization of the findings regarding the construct, future studies could replicate this study in nonprofit sectors in other countries/regions. Moreover, it would be advantageous for future research to use samples in different sectors (i.e., commercial sector and government sector) to determine if the results concerning the influencing mechanism hold. Second, the causal relationship between the model variables should be carefully interpreted due to the usage of a cross-sectional design. Therefore, a longitudinal study may offer the opportunity to clearly identify the causality among the examined variables. Third, all examined measures are self-reported and common method bias might exist, although the CFA results showed that this was not a major issue. Future studies should further mitigate the concern over common method bias by collecting data from different sources, such as supervisors and peers. Fourth, although our study identified a chain mediating effect of POF and IntSB on the internal branding–brand performance relationship, there might be some individual or contextual factors that strengthen or buffer this mediation effect. Therefore, we encourage further research to investigate the boundary conditions in the proposed model.

## Data Availability Statement

The raw data supporting the article conclusion will be made available on the request from the corresponding author.

## Author Contributions

RZ conceived of the idea and methods, and drafted the manuscript. YW was involved in the manuscript design and charge of the manuscript revision. CY helped in collecting data, analyzed the results, and drafted the part of the literature review. All authors contributed to the article and approved the submitted version.

## Conflict of Interest

The authors declare that the research was conducted in the absence of any commercial or financial relationships that could be construed as a potential conflict of interest.

## Publisher’s Note

All claims expressed in this article are solely those of the authors and do not necessarily represent those of their affiliated organizations, or those of the publisher, the editors and the reviewers. Any product that may be evaluated in this article, or claim that may be made by its manufacturer, is not guaranteed or endorsed by the publisher.
